# Campylobacter Infection Introduced Following Fecal Microbiota Transplantation

**DOI:** 10.7759/cureus.62541

**Published:** 2024-06-17

**Authors:** Brian R Beyer, Cody Sheppard, Jordyn Mullins, Anthony Igbadumhe

**Affiliations:** 1 Medicine, Burrell College of Osteopathic Medicine, Las Cruces, USA; 2 Family Medicine, Burrell College of Osteopathic Medicine, Las Cruces, USA

**Keywords:** recurrent c. difficile, screening guidelines, clostridium difficle infection, fecal microbiota transplant, campylobacter infection

## Abstract

Fecal microbiota transplantation is an evidence-based therapeutic option for recurrent *Clostridium difficile* infection, involving the transfer of healthy donor fecal material to restore gut microbial balance. Despite meticulous donor screening, *Campylobacter jejuni*, a prevalent cause of bacterial gastroenteritis, is not routinely tested, potentially impacting fecal microbiota transplant safety. We present a case of a female with recurrent *C. difficile* infection treated with fecal microbiota transplantation, complicated by a subsequent *C. jejuni* infection. The emergence of *Campylobacter* post fecal microbiota transplantation underscores the importance of comprehensive donor screening protocols. Our case prompts a reevaluation of fecal microbiota transplantation safety measures and advocates for inclusive screening to enhance patient outcomes.

## Introduction

Fecal microbiota transplantation was first implemented in humans in 1958, although it has been practiced in veterinary medicine for over 100 years, particularly in horses with intractable diarrhea [1]. It is now an evidence-based management for patients with recurrent or treatment-resistant *Clostridium difficile *infection. This procedure involves transferring fecal material from a healthy donor to a patient's gastrointestinal tract to restore disrupted microbial balance [1,2]. Despite meticulous screening of donors, which includes initial health questionnaires to determine eligibility and subsequent blood and stool testing for various pathogens, colonization of the recipient with harmful pathogens such as multi-drug-resistant *Escherichia coli*, *Campylobacter jejuni*, and other enteropathic organisms continues to occur post-fecal microbiota transplantation [3]. This highlights the need for standardized screening and laboratory protocols for potential donors [4].

*Campylobacter* spp. are gram-negative bacteria with roughly 17 subspecies commonly found in reservoirs such as contaminated poultry, red meat, unpasteurized milk, vegetables, bird feces, sewage, and drinking water [5,6]. As an obligate microaerophile, *Campylobacter *resides in the gastrointestinal tract where there is minimal oxygen exposure, with most colonization occurring in the cecum. Growth is often invasive, resulting in damage to enterocytes and leading to bloody gastroenteritis [7]. Notably, *C. jejuni* is sometimes overlooked in routine screenings, creating a potential gap in fecal microbiota transplantation safety protocols. Our case report adds a critical dimension to the existing literature by presenting a compelling case of recurrent *C. difficile* infection treated with fecal microbiota transplantation, complicated by a subsequent *C. jejuni* infection. This case prompts a reevaluation of screening protocols for fecal donors, underscoring the need for comprehensive assessments encompassing a broader spectrum of potential pathogens.

In the following sections, we will delve into the details of the case, exploring the patient's clinical history, the fecal microbiota transplantation procedure, and the subsequent challenges posed by the emergence of *C. jejuni *infection. By examining this case, we aim to contribute valuable insights to the evolving landscape of fecal microbiota transplantation safety and advocate for a more inclusive screening process to enhance patient outcomes.

## Case presentation

A 51-year-old female presents with a past medical history of diabetes mellitus of six years duration managed with insulin, chronic kidney disease of four years duration managed with blood pressure control via lisinopril 20 mg daily, and multiple recurrent *C. difficile* infection that started eight months ago which were refractory to extended courses of vancomycin and fidaxomicin. The first episode was managed with vancomycin 125 mg orally four times daily for 10 days. She had a recurrence three months later which was managed with fidaxomicin 200 mg orally twice daily for 10 days. She had a second recurrence six months after her initial presentation which was managed again with fidaxomicin 200 mg orally twice daily for 10 days. The patient had a third recurrence one day ago which was managed with fecal microbiota transplantation.

The donor feces were initially screened for multiple pathogens including* C. difficile*, *Entamoeba histolytica*, *Blastocystis hominis*, *Dientamoeba fragilis*, *Giardia lamblia*, *Cryptosporidium spp*, *Cystosospora belli*, *Cyclospora cayetanensis*, *Microsporidia* and *Helicobacter pylori *fecal antigen. Then the feces were subsequently mixed with sterile saline, filtered, and introduced to the terminal ileum through a colonoscope. Initially, there was marked improvement in symptomatology; however, one day later, the patient began experiencing symptoms like the prior infection. As her symptoms of watery diarrhea persisted, she remained hospitalized, and a rapid* C. difficile* fecal antigen test revealed a recurrence of *C. difficile* infection. At this time no other gastrointestinal pathogens were tested.

**Figure 1:**
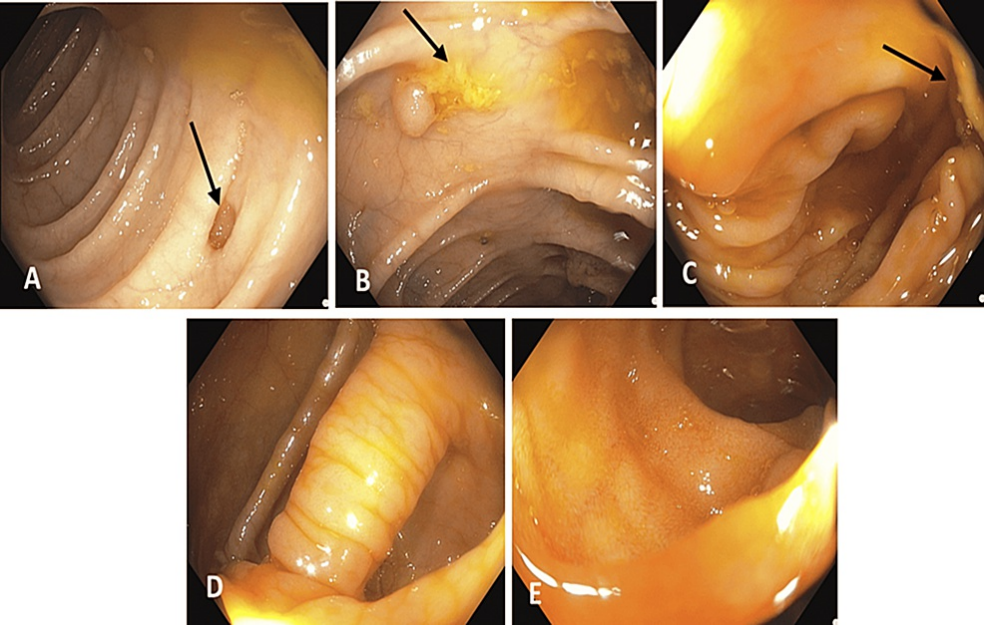
Colonoscopy for evaluation of recurrent Clostridium difficile (A) Colonic diverticula; (B) Pseudo-membrane formation; (C) Additional pseudo-membrane formation; (D/E) Generally unremarkable colonoscopy

**Figure 2:**
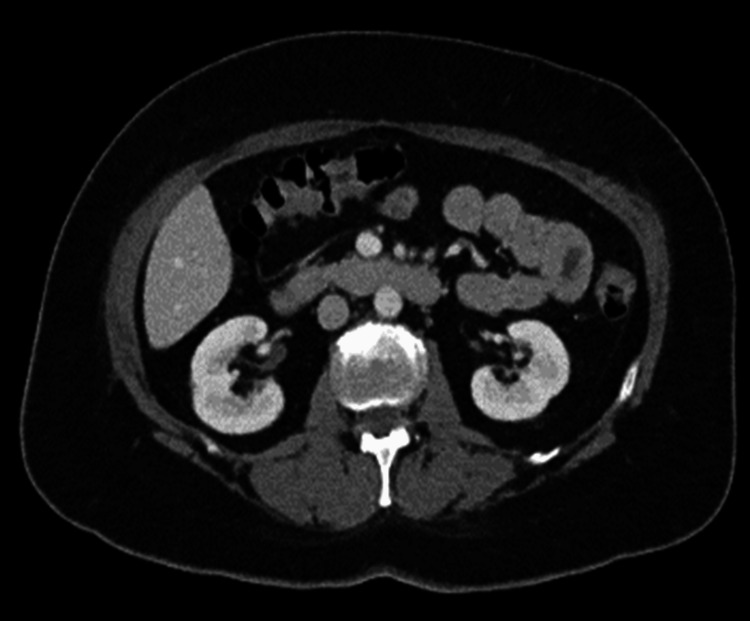
Computed tomography scan on recent admission showing no acute intraabdominal process with no inflammatory changes of the colon. General evidence of cholelithiasis, hepatic steatosis, and colonic diverticulosis were noted.

She was initially managed with vancomycin 125 mg orally once daily and was put on a soft foods diet. However, after four days of treatment, the patient continued to experience severe watery diarrhea and abdominal pain, particularly in the left lower quadrant, described as severe and non-radiating. Imaging studies such as colonoscopy (Figure 1) and computed tomography scan (Figure 2) showed fatty liver and possible gallbladder sludge. Additionally, the patient exhibited signs of malabsorption secondary to chronic diarrhea and weight loss. Additional testing revealed minor neutropenia, most likely due to intractable nausea, vomiting, diarrhea, and nutritional deficits. Further diagnostic evaluation, including a gastrointestinal bacterial panel that tested for *Campylobacter* species, *C. difficile*, *Plesiomonas shigelloides*, *Salmonella species*, *Vibrio species*, *Yersinia species*, *E. coli*, *Shigella*, *Cryptosporidium *species, *C. cayetanensis*, *E. histolytica*, *G. lamblia*, *adenovirus*, *astrovirus*, *norovirus*, *rotavirus*, and *sapovirus*. Results showed a resolution of *C. difficile*, but a new *C. jejuni* infection. No other contacts had evidence of *C. jejuni* infection.

Per the consulting gastroenterologist's recommendations, antibiotics were withheld for 48 hours to preserve the fecal transplant and observe if the *C. jejuni* infection would resolve spontaneously. The patient also received dicyclomine 40 mg four times daily for abdominal cramping and hydrocortisone suppository 25 mg rectal twice daily. Within two days, the patient's symptoms improved with supportive care. After three more days of supportive measures, symptom resolution was observed. Nutritional deficits were corrected via supplementation with folic acid, iron, and B12 from a prenatal vitamin. Labs including complete blood count indicated resolving leukocytosis, and the patient's diarrhea was subsiding as she had two formed stools. The patient was cleared for discharge and sent home on a prenatal vitamin, dicyclomine 40 mg as needed, and hydrocortisone suppository 25 mg rectal twice daily for one more week. She also restarted her home medications at the time of discharge. On follow-up two weeks later, repeat stool infectious studies showed complete resolution of the patient’s symptomatology and *C. jejuni* infection.

## Discussion

The emergence of *C. jejuni* infection in the context of fecal microbiota transplantation presents a notable and previously underrecognized complication. Our case, consistent with findings in hypogammaglobulinemia patients [8], underscores the significance of thorough screening protocols for potential pathogens in fecal donors. Currently, practices for stool donors are unstandardized and largely based on joint society consensus recommendations [3,4,9]. These recommendations include donor eligibility, methods, and materials for stool collection/preservation, and laboratory testing. While these recommendations are strict, there are no existing guidelines to comprehensively address donor-to-recipient transmission of infections.

The patient in our case underwent comprehensive testing for common gut pathogens before the fecal microbiota transplantation procedure, yielding negative results for *C. jejuni*. This pre-fecal microbiota transplantation negativity is intriguing and leads us to hypothesize that the patient's susceptibility to *C. jejuni* may have been heightened by her underlying neutropenic state. The compromised immune state may have played a key role in the failure of protection against bacterial pathogens like *C. jejuni* and *Clostridia* species. This could explain the patient's history of recurrent *C. difficile* infections prior to the fecal microbiota transplantation.

An essential aspect of our case is the timing of* C. jejuni* colonization post-fecal microbiota transplantation, suggesting a potential correlation between the transplantation and the subsequent emergence of the pathogen. The ability of *C. jejuni* to form biofilms on mucosal surfaces of the intestines may contribute to its antimicrobial resistance, providing a plausible mechanism for its colonization after fecal microbiota transplantation in a susceptible host [8,10,11]. This phenomenon highlights the need for heightened vigilance in assessing the risk factors and immune status of recipients, especially in the setting of known predisposing conditions.

Traditionally, *Campylobacter* infections are not treated with antibiotics and the disease is generally self-limiting such as in our case. However, for severely ill patients symptoms include bloody stools, high fever, extraintestinal infection, and symptoms lasting more than seven days [12]. The implications of *C. jejuni* colonization in the bowels are far-reaching, predisposing the patient to severe complications, including bloodstream infections. Given the patient's history of recurrent *C. difficile* infections, prompt and effective eradication of *C. jejuni* from the intestinal and bloodstream becomes paramount.

## Conclusions

Our case report sheds light on a crucial aspect of fecal microbiota transplantation safety, urging the standardization of screening protocols for fecal donors. Specifically, the inclusion of *C. jejuni *in routine screenings is advocated, particularly in patients with hypogammaglobulinemia, neutropenia, or other immunocompromised states. The interplay between the patient's immune status, the fecal microbiota transplantation procedure, and the subsequent *C. jejuni* infection raises pertinent questions about the dynamics of gastrointestinal microbiota and its potential impact on susceptible hosts.

In conclusion, our findings accentuate the need for a more comprehensive approach to fecal microbiota transplantation safety, considering the nuanced interplay between the recipient's immune status and the risk of emerging pathogens. By incorporating robust and universal screening measures and tailoring interventions based on individual patient profiles, we can enhance the safety and efficacy of fecal microbiota transplantation procedures, ensuring optimal outcomes for patients undergoing this innovative therapeutic strategy.
